# Common Pitfalls in the Interpretation of Endocrine Tests

**DOI:** 10.3389/fendo.2021.727628

**Published:** 2021-09-07

**Authors:** Jose C. Alvarez-Payares, Jesus David Bello-Simanca, Edwin De Jesus De La Peña-Arrieta, Jose Emilio Agamez-Gomez, Jhon Edwar Garcia-Rueda, Amilkar Rodriguez-Arrieta, Luis Antonio Rodriguez-Arrieta

**Affiliations:** ^1^ Internal Medicine Department, Faculty of Medicine, University of Antioquia, Medellin, Colombia; ^2^ Internal Medicine Service, Institución Prestadora de Servicios (IPS) Universitaria - Clínica León XIII, Medellin, Colombia; ^3^ Faculty of Medicine, University of Antioquia, Medellin, Colombia; ^4^ Faculty of Medicine, University of Cartagena, Cartagena, Colombia; ^5^ Endocrinology Section, Internal Medicine Department, Faculty of Medicine, University of Antioquia, Medellin, Colombia

**Keywords:** endocrine test, hook effect, hyperprolactinemia, adrenal insufficiency, Cushing's syndrome, acromegaly, hypogonadism

## Abstract

Endocrine tests are the cornerstone of diagnosing multiple diseases that primary care physicians are frequently faced with. Some of these tests can be affected by situations that affect the proper interpretation, leading to incorrect diagnoses and unnecessary treatment, such as the interference of biotin with thyroid function test, falsely elevated prolactin values in presence of macroprolactinemia or falsely normal due to the “hook effect” in macroprolactinomas. Recognizing these situations is essential for the clinician to make an adequate interpretation of these tests as well as an accurate diagnosis that guarantees the best outcomes for the patient.

## 1 Introduction

Despite the development of laboratory techniques in the last few years, problems and errors occurring while interpreting endocrine tests persist. These errors lead to misinterpretations during the initial clinical impression and therefore, the therapeutic approach. This article reviews the most common errors while interpreting endocrine tests' including the hook effect and falsely normal values of prolactin (PRL) mainly in macroprolactinomas, macroprolactinemia and falsely elevated PRL, macro thyrotropinoma and falsely elevated thyroid-stimulating hormone (TSH) levels, heterophile antibodies leading to a false hormone elevation, biotin interference with hormonal assays and cross-reactivity of steroidal hormones with immunoassays cases and a brief overview of the other conditions.

Additionally, because of the large number of endocrinology laboratory tests available, we outline a brief description of the laboratory tests that are most frequently used to diagnose the disorders in each of the disease groups, with an emphasis on when to request such tests, how to interpret them, the most common errors that occur during interpretation, typical clinical scenarios, and recommendations to avoid these errors for each axis. The objective of this article is to present a useful tool for the general and primary care physician who treats patients with endocrine disorders on a day-to-day basis to have a better understanding the laboratory tests used in the diagnosis of the more commonly encountered disorders.

## 2 Lactotroph Axis

### 2.1 When to Request a Lactotroph Axis Test? 

A lactotroph axis test should be requested when the clinical presentation suggests axis' hyperfunction or hypofunction. Hyperfunction in men is suspected when characteristics, such as libido reduction, gynecomastia, impotence and galactorrhea are present. In premenopausal women, hyperfunction can be suspected when a patient presents with oligomenorrhea or amenorrhea, galactorrhea and libido reduction. For postmenopausal women, hyperfunction should be suspected when the tumors' mass effect is predominant at the same time it is important to note that, the absence of galactorrhea blurs the diagnosis. Hypofunction in women can be suspected if they present with breastfeeding difficulties. No clinical syndrome for hypofunction is observed in men or non-pregnant women (1).

### 2.2 Which Tests Must Be Ordered? 

Serum PRL is recommended for diagnosis. A single PRL level is usually adequate to diagnose hyperprolactinemia. Nonetheless, when the diagnosis is unclear, the test must be repeated.

### 2.3 How to Interpret These Tests? 

An elevated PRL level is diagnostic as long as the venous sample was obtained without excessive stress. Most centers consider normal values of < 25 ng/ml in women and < 20 ng/ml in men. A PRL level > 500 ng/ml is virtually diagnostic for a prolactinoma but drug-induced hyperprolactinemia must be excluded (1, 2).

### 2.4 Main Mistakes in the Interpretation and Recommendations to Avoid Mistakes 

#### 2.4.1 Falsely Normal Values (Hook Effect)

**Figure 1:**
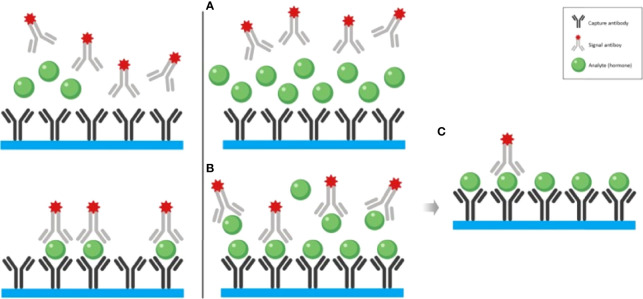
The left panel illustrates the non-competitive “sandwich” immunoassay with normal (or elevated within the tolerance of the assay kit) hormone concentration. The right panel illustrates the mechanism of the hook effect with exceedingly high hormone concentration. **(A)** At the sample that contains remarkably elevated hormone concentration is added to the test tube which contains both capture and signal antibodies. **(B)** It is both the capture and signal antibodies preventing the formation of the “sandwiches”. **(C)** After the washout phase, only a few “sandwiches” will be left producing a low signal. Adapted from Haddad et al. Clinical Diabetes and Endocrinology (2019) 5:12 (3) with previous authorization from the author (2).

When a large pituitary adenoma is associated with a slightly elevated PRL levels in immunoassays, a 1:100 serum dilution is recommended in order to exclude the Hook effect (3, 4). This phenomenon occurs with immunoassays, particularly with a two-site (sandwich) non-competitive monoclonal immunoassay, where the excess of antigens (PRL) saturates antibodies and avoids the capture and sandwich formation, leading to an erroneously low result (5) (**Figure 1**).

#### 2.4.2 Falsely Elevated Values

A PRL level > 500 ng/ml is virtually diagnostic for a prolactinoma but drug-induced hyperprolactinemia must be excluded (1–4). PRL circulates in the blood in three molecular forms, a) small PRL of 23 kilodaltons (kDa) (90% of serum PRL), b) big PRL of 45-50 kDa, and c) big-big PRL of > 100 kDa (<1% of serum PRL). Big-big PRL is responsible for macroprolactinemia when large molecular weight PRL is the predominant form in the serum (5). Macroprolactin interferes with most immunoassays, leading to a falsely elevated PRL (3). It is not biologically active due to its low affinity with PRL receptors and does not provide negative hypothalamic feedback. Therefore, true hyperprolactinemia can elevate even further (monomeric hyperprolactinemia) (6). Macroprolactinemia must be considered especially when no hyperprolactinemia symptoms such as galactorrhea, oligomenorrhea/amenorrhea, headache, or visual impairment are present. However, it is important to note that the presence of these symptoms - galactorrhea, menstrual disorders, or infertility have been reported in up to 45% patients with macroprolactinemia, hence should not exclude this diagnosis (7). Approximately 10% of monomeric hyperprolactinemia patients may be asymptomatic. In cases with symptoms and a confirmed macroprolactinemia, pituitary mass must be ruled out (8). In doubtful cases, the test must be repeated on a different day, ideally with two blood samples with a 15–20 minute difference, to avoid stress-associated PRL pulsatility.

Differential diagnoses must be taken into account: pregnancy is the most common cause of hyperprolactinemic amenorrhea. Drugs are the most common cause of non-tumoral hyperprolactinemia with antipsychotics (typical and atypical) being the most frequently associated, followed by prokinetics such as metoclopramide and domperidone. The incidence among patients using selective serotonin reuptake inhibitors is less clear, as is the role of conjugated oral contraceptives (1, 2). Physiological causes of hyperprolactinemia should be taken into account as well (physical activity and postcoital among others). In asymptomatic patients with hyperprolactinemia, to avoid macroprolactinemia mistakes, multiple tests can be performed. Gel filtration chromatography (GFC) is the gold standard test to differentiate between different molecular weight prolactins (6), however, this method is both time and labor-intensive. Therefore, an alternative technique (PEG precipitation test) has become the most used one (9). In PEG precipitation test, macroprolactin is precipitated and monomeric prolactin is left in the supernatant. Macroprolactinemia is usually suspected when precipitable prolactin with PEG exceeds 60% of the total prolactin or when monomeric prolactin in the supernatant is less than 40% of total prolactin (2, 10).

#### 2.4.3 Functional Hyperprolactinemia in PCOS

The elevation of PRL in PCOS is explained due to the presence of macroprolactin, it is therefore essential to screen it. This first step is crucial to avoid a misdiagnosis and to spare an unnecessary pituitary magnetic resonance imaging (MRI) or even a dopaminergic agonist treatment (5, 8).

## 3 Corticotropic Axis

### 3.1 When to Request a Corticotropic Axis Test Once Hypo or Hyperfunction Is Clinically Suspected

#### 3.1.1 Hypofunction Suspicion

The clinical spectrum of adrenal insufficiency (AI) is variable, based on the onset (acute, subacute, chronic, or an adrenal crisis). This test must be considered in any patient presenting with vasodilatory shock and isolated deficiency of adrenocorticotropic hormone (ACTH) which although rare, it should be considered in patients with persistent asthenia and hypoglycemia trends. AI is a disease with slow onset and usually manifests within physical stress or concomitant to severe diseases. Clinical presentation includes asthenia, anorexia, gastrointestinal symptoms (nausea, vomiting, abdominal pain) (11). Physical examination shows hypotension as the most prevalent sign. In primary AI, skin and mucosa hyperpigmentation is observed [due to ACTH hypersecretion and therefore, pro-opiomelanocortin (POMC)] which is visible in palms, soles, scars, and mucosa. Hyponatremia and hyperkalemia are directly dependent on the mineralocorticoid deficiency and are present in primary forms only (mineralocorticoid and glucocorticoid deficiency), not in central forms (12).

#### 3.1.2 Hyperfunction Suspicion

Hypercortisolism has plenty of clinical manifestations, but none of them is pathognomonic. From most to less common ones are obesity or weight gain, facial plethora, irregular menses, hirsutism, acne, arterial hypertension, proximal myopathy, capillary fragility, scarring disorders, red-wine striae, psychiatric disorders, carbohydrate intolerance/diabetes mellitus, osteoporosis, increased risk of infectious diseases, nephrolithiasis due to hypercalciuria, and hyperpigmentation (12).

Cushing's syndrome must be suspected in young adults with osteoporosis and/or hypertension, difficult-to-control diabetes, pediatric patients with growth delay, weight gain, and adrenal incidentalomas. Of all symptoms facial plethora, fragile capillary, bruising with minimal trauma (≥3 ecchymoses of > 1 cm in diameter), proximal muscular weakness, and red striae bigger than 1cm are more specific to the diagnosis of Cushing's syndrome. It is important to note that a skin-fold thickness < 2mm has a positive likelihood ratio (LR+) of 116 for Cushing's syndrome diagnosis (13). According to European criteria, Cushing's syndrome work-up should be performed in the following cases: 1. weight loss with central obesity. 2. uncommon signs or symptoms for the respective age such as osteoporosis and arterial hypertension. 3. multiple signs or symptoms suggestive of Cushing's syndrome. 4. overweight children with short height 5. adrenal incidentaloma (14).

### 3.2. Which Tests Should Be Requested?

#### 3.2.1 Hypofunction

Three pillars are the basis for the diagnosis. These include confirming the existence of AI (syndromic diagnosis), identifying the location of the responsible defect (localization diagnosis), and identification of the cause (etiological diagnosis). The following laboratory tests are covered in this review:

- Basal serum cortisol: Can confirm or rule out the disease without any additional tests, therefore it should be the first test that is performed.
- Salivary cortisol: When performing this test, physicians must ensure that the sample should be drawn between 8:00 and 9:00 hours.
- Plasmatic ACTH: This test is fundamental to distinguish adrenal forms from hypothalamic-pituitary forms.
- Dynamic tests: Dynamic tests are required to perform syndromic diagnosis when basal serum cortisol has an intermediate value. These include ACTH stimulation test that is the most widely available. CRH stimulation test that has lower standardization and availability and insulin-induced hypoglycemia test which is the gold standard test for AI diagnosis., However, complications have limited insulin-induced hypoglycemia test's applicability. The use of dynamic tests with metyrapone or glucagon stimulation are not standardized and therefore are no longer recommended.

a) ACTH Stimulation test: Stimulation test with a 250 ug IV or IM tetracosactide (Synacthen^®^) with cortisol sampling at zero, 30, 60 minutes is a simple and secure way to evaluate adrenal function. Both post-stimulation cortisol values must be > 18 ug/dL to rule out a primary AI (15). The common mistake is that this test with high ACTH analog doses does not rule out primary AI or central AI. Therefore, in these scenarios, a low dose tetracosactide (Synacthen^®^) stimulation test (1ug/IV) should be conducted, since better diagnostic performance has been demonstrated to confirm central AI (16).

#### 3.2.2 Hyperfunction

When hyperfunction is detected the following are first-line tests:

- 24-hour urinary free cortisol (UFC): While conducting this test two measurements should be performed for more accurate results.
- Low dose dexamethasone suppression test (LDDST): Under this test, two types of tests are available, these include:

a) Overnight Screening Test: This test is performed by administering 1 mg of dexamethasone orally at 23:00 hours and measuring plasma cortisol at 08:00 the next day and then 0.5 mg every 6 hours for 48 hours. Generally, it's reserved for doubtful cases, such as pseudo-Cushing (9) and;

b) Late night salivary cortisol: This test can confirm the diagnosis of Cushing's syndrome and at least two measurements should be performed. It is important to note that the interpretation criteria for this test isn't standardized due to differences in methodology (17).

- Nocturnal serum cortisol: This test evaluates cortisol's circadian rhythm and it requires hospital admission.
- Loperamide suppression test: Although recent studies of this method are scarce, this test is performed by obtaining samples of serum cortisol at 0, 150, 180 and 210 min (18). It is interesting to note that some conditions such as anticonvulsant treatment, depression, etc. can cause false positives in the LDDST. Therefore, in such cases, a dose of oral loperamide 0.15-0.20 mg/kg (16mg maximum) could be administered to prevent a false positive.
- Plasmatic ACTH: This test is performed after Cushing's syndrome diagnosis is confirmed to determine the etiology and identify if hypercortisolism is ACTH dependent (suggestive of a pituitary or ectopic origin) or ACTH independent (suggestive of an adrenal origin).

### 3.3 How Are These Tests Interpreted?

#### 3.3.1 Hypofunction

- Basal serum cortisol: In this test, normal values refer to samples drawn between 8:00 and 9:00 hours due to the circadian dynamic of cortisol secretion leading to a variation in diagnoses. Diagnosis is confirmed when cortisol values are <3 ug/dL and have a 100% sensitivity. Diagnosis is considered highly suggestive when cortisol values range between 3-5 ug/dL and some authors consider that no additional tests are required. Cortisol values between 5-15 ug/dL are considered a gray zone where confirmatory tests could prove useful. AI is ruled out when cortisol values > 15 ug/dL (other authors recommend > 18 ug/dL) (19).
- Salivary cortisol: This test should be requested when an artifact is hampering a basal cortisol test interpretation. It has been widely accepted that if a morning (8:00 hours) concentration of salivary cortisol is > 0.58 ug/dL (16 nmol/l) AI is ruled out and salivary cortisol values of < 0.18 ug/dl (5 nmol/l) is strongly predictive of AI.
- Plasmatic ACTH: In primary AI (adrenal involvement), reduced cortisol levels lead to an increased ACTH secretion, which generate plasmatic ACTH values up to double the normal levels. In secondary AI (pituitary involvement), ACTH values are low or inappropriately normal for the low cortisol levels. In tertiary AI (hypothalamic involvement, i.e. abrupt cessation of glucocorticoid therapy) CRH secretion is altered and therefore, ACTH (with low values) as well.
- Dynamic tests: See **Table 1**.

**Table 1:** Dynamic tests for the study of adrenal function.

Test name	Compound to administer	Sampling	Test	Observations	Interpretation
Insulin hypoglycemia (IH)	Regular insulin 0.1-1.15 IU/kg weight i.v.	0-30-45-60-90 min	Serum Cortisol	Gold Standard Evaluates the integrity of the entire HHA axis Contraindicated in age> 60 years, cardiovascular disease or cerebrovascular, severe hypertension, pregnancy Assessable if blood glucose <40 mg /dl	Normal: maximum cortisol value> 18 μg/dl
Stimulation with ACTH in standard doses	Tetracosctide (Synacthen®) 250 mg i.v.	0-30-60 min.	Serum Cortisol	Safe and Simple. Assess adrenal function	Normal: maximum cortisol value> 18 μg/dl
Low-dose ACTH stimulation	Tetracosctide (Synacthen®) 1 mg i.v.	0-30-60 min.	SerumCortisol	Safe and Simple. Assess function of the adrenal axis	Normal: maximum cortisol value> 18 μg/dl
Methopyrone stimulation	Methopyrone 30 mg / kg v.o.	8 h postmethopyrone	11-deoxycortisol	Evaluates the integrity of the HHA axis Alternative to IH when contraindicated	Normal: 11-deoxycortisol> 7 μg / dl, 8 h after the administration of methopyrone in the presence of cortisol values <5 μg/dl
Glucagon stimulation	Glucagon 1 mg i.m	90-120-150-180- 210-240 min.	Serum Cortisol	Evaluates the integrity of the HHA axis Lower diagnostic precision than the previous ones	Normal: maximum cortisol value> 21.58 μg / dl

ACTH, corticotropin; HHA, hypothalamic-pituitary-adrenal; HI, insulin hypoglycemia; HT, arterial hypertension; IS, adrenal insufficiency; i.m., intramuscular; i.v., intravenous; v.o., orally. Adapted from de Miguel Novoa et al. (19, 20).

#### 3.3.2 Hyperfunction

- A Cushing's syndrome diagnosis will be confirmed when at least two first-line (screening) tests are positive (21).
- To improve the diagnostic sensitivity of screening test, the upper reference value of > 145 ng/dL for salivary cortisol is used. For UFC, three times the upper reference value and plasmatic 8:00 hours cortisol > 1.8 mcg/dL after 1 mg dexamethasone administered the previous day is used. These cutoffs improve sensitivity (Sens), reduce the specificity (Spe), and additional confirmatory tests are required to rule in or rule out the diagnosis.
- If the screening test are normal in patients with low clinical suspicion, it is unlikely the disease is present unless mild or cyclical hypercortisolism is present. If two negative results in two different tests are observed, no other tests are recommended (21).
- Nocturnal serum cortisol: A value of > 7.5 mcg/dL is necessary for a Cushing's syndrome diagnosis (Sens = 96%, Spe = 100%). A value of < 1.8 mcg/dL excludes the diagnosis.
- For the etiological diagnosis, the plasmatic ACTH value must be evaluated. This value is usually between 20-80 pg/mL at 8:00 hours decreasing during the day to 20 pg/mL at 16:00 hours with a further decrease to less than 5-10 pg/mL one hour after sleep begins. Nevertheless, if pathological hypercortisolism is present, the circadian rhythm is lost (12). Therefore, if a patient with hypercortisolism presents with low ACTH values (less than 5 pg/mL), it indicates that it is ACTH independent, and an imaging study should be performed (adrenal protocol CT or MRI) and in case of evidence of adrenal lesions, surgical or medical therapy must be considered. Conversely, when hypercortisolism is ACTH dependent, the results usually are above 20 pg/mL, which indicates a pituitary or ectopic origin. Intermediate ACTH levels between 5-20 pg/mL are ambiguous or indeterminate and usually indicate ACTH-dependent cortisol secretion. In these cases, it is recommended to request a CRH stimulation test. The CRH stimulation test shows a complete lack of response if it is of adrenal origin or on the other side, if there is an elevation of ACTH, a diagnosis of ACTH-dependent Cushing’s syndrome may be considered (22).
- In cases of ACTH-dependent hypercortisolism, a pituitary cause should be initially evaluated; studies are aimed at identifying a possible corticotropic adenoma with MRI or performing an inferior petrosal sinus sampling (IPSS) (21, 23). If ACTH production is ectopic, studies should be performed to identify the etiology, such as: CT chest, abdomen, pelvis, or consider gallium-68 DOTATATE scan or, depending on availability, 18 F-Dopa positron emission tomography PET-CT. An alternative approach in cases of ACTH-dependent hypercortisolism is to perform the SSDT at 11:00 p.m. at high doses with 8 mg of dexamethasone - 80% of pituitary tumors will be suppressed and will present a decrease of greater than 50% in subsequent cortisol. At these doses, however, up to 20% of cases of ectopic ACTH-producing tumors are also suppressed with high doses of dexamethasone, so this strategy before performing pituitary MRI or IPSS will depend on the protocols and availability of these studies in each institution (23).

### 3.4 Main mistakes in the Interpretation and Recommendations to Avoid Mistakes

- In pregnancy, 24-hour urinary cortisol or late-night salivary cortisol is preferred. In adrenal incidentaloma, a 1 mg dexamethasone test is preferred. In cyclical Cushing's syndrome hypercortisolism episodes alternate with normal ones, so urinary cortisol and late-night salivary cortisol should be conducted during the dexamethasone suppression test. If the initial test is normal and clinical suspicion persists, tests should be repeated during follow up. In patients with chronic kidney disease with glomerular filtration rate less than 30 ml/minute, 1 mg dexamethasone suppression test is recommended over urinary cortisol (**Table 2**). In patients with epilepsy, urinary cortisol or salivary cortisol is recommended, as many anticonvulsants increase dexamethasone clearance (12, 21).
- The main protein involved in cortisol transport is the cortisol binding globulin (CBG). Methods used for cortisol measurement do not distinguish protein-bound cortisol (90%) from free cortisol (10%). Therefore, plasmatic cortisol values should be interpreted carefully in situations that could alter CBG concentrations (21, 23).
- This way, if a reduction in CBG synthesis occurs (liver disease, hypothyroidism, sepsis) or if renal losses are increased (nephrotic syndrome), a falsely low cortisol level may be found. Conversely, cortisol levels will be falsely elevated when an increase in CBG synthesis occurs (hyperthyroidism, pregnancy, estrogen treatment). This artifact may be avoided if salivary cortisol is measured, as it presents an adequate correlation with serum cortisol levels and isn't modified by CBG concentration (21, 23).
- Night serum cortisol is recommended to be drawn in the first two inpatient days to avoid an elevation related to hospitalization-induced stress.
- A low-dose dexamethasone suppression test should not be performed in pregnant patients or conditions in whom abnormal CBG concentrations may be found such as estrogen intake (increases CBG) or liver disease (decreases CBG) (**Table 2**). In the case of estrogen intake, it should be suspended, and a new measurement should be performed in four to six weeks.
- False positives such as physiological hypercortisolism (also known as pseudo-Cushing) could occur, as this rarely elevates cortisol levels above the cutoff for diagnosis. Dynamic tests such as low dose dexamethasone suppression test allow to rule out the excessive endogenous liberation of cortisol (21, 22).
- A single normal result cannot rule out the diagnosis, specifically in patients with intermittent (cyclical) hypercortisolism. Additional tests should be performed to confirm the diagnosis (24).
- False-positives in measurement of UFC levels may be observed in diuresis greater than 3 liters, pregnancy, glomerular filtration rate less than 30 ml/min, and women with significant weight loss after bariatric surgery (**Table 2**) (24).
- Evaluation in critical patients: Inadequate cortisol levels in the context of acute stress may be observed in what is known as critical illness-related corticosteroid insufficiency (CIRCI). Whenever CIRCI is suspected, cortisol levels must be evaluated, and ACTH measurement should be requested before glucocorticoid therapy is initiated. A frequent mistake when this condition is suspected is that glucocorticoid therapy is started without a cortisol measurement, therefore affecting the axis and interfering with further laboratory analysis. A cortisol level < 10ug/dL is highly suggestive of CIRCI and no further studies are needed to confirm the diagnosis. In cases of doubt due to cortisol level > 10 ug/dL, a dynamic test is recommended and a 30-minute delta cortisol post-ACTH should be calculated, however, this possibility is limited. Additionally, whenever the doubt exists due to cortisol values of > 10 ug/dL, therapy should be individualized and a new evaluation should be performed after the critical state is overcome to rule in or rule out AI (12).
- When the patient is under prednisolone treatment for any reason, the cross-reactivity of the active molecule with cortisol immunoassays must be considered. This is the reason liquid chromatography-mass spectrometry (LC-MS) has become such a popular technique for such cases, as it provides an accurate measurement of steroidal hormones to overcome the crossreactivity previously mentioned. The medication could be swapped to synthetic steroids that do not have cross-reaction such as dexamethasone (25).

**Table 2:** Pitfalls in the interpretation of the dexamethasone suppression test and 24-hour urinary free-cortisol excretion.

Dexamethasone suppression test	24-hour UFC excretion
False-positive tests (i.e., lack of suppression)	Drugs/conditions that increase UFC
Non-Cushing hypercortisolemia	- Exercise/stress
- Obesity	- Proteinuria
- Stress	- Carbamazepine (if measured by HPLC)
- Alcoholism	- Fenofibrate (if measured by HPLC)
- Psychiatric illness (anorexia nervosa, depression, mania)	- Some synthetic glucocorticoids (immunoassays)
- Elevated cortisol binding globulin (estrogen, pregnancy, hyperthyroidism)	Conditions that decrease UFC
- Glucocorticoid resistance	- Incomplete collection
Test-related artifacts	- Low glomerular filtration rate
- Laboratory error, assay interference	- Urinary tract infection
Insufficient dexamethasone delivery into the circulation	
- Non compliance	
- Decreased absorption: for instance, bowel resection	
- Increased metabolism (drugs): phenobarbital, phenytoin, carbamazepine, topiramate; Nifedipine; among others.	
- Decreased metabolism (drugs): itraconazole, ritonavir, fluoxetine, diltiazem, among others.	
False-negative tests	
- Chronic renal failure (creatinine clearance < 15 mL/min)	
- Hypometabolism of dexamethasone (e.g., liver failure)	

HPLC, high-pressure liquid chromatography.

## 4 Somatotropic Axis

### 4.1 When to Request a Somatotropic Axis Test?

*a) Hypofunction Suspicion:* There are several groups of adult patients who are at risk for GH deficiency, including those with history of sellar mass lesions, pituitary surgery or radiotherapy, traumatic brain injury, subarachnoid hemorrhage, and childhood onset GH deficiency. Patients in these groups are generally at risk for additional pituitary hormone deficiencies; idiopathic GH deficiency of adult onset is extremely unlikely to exist (26). It is for this reason we will not deepen in this topic. However, it must be mentioned that GH levels are typically undetectable between secretory spikes in healthy adults. As a consequence, random GH levels are of no diagnostic utility in the evaluation of GH deficiency. The diagnosis of GH deficiency generally rests on demonstrating lack of GH secretory response to one of several pharmacologic agents that normally trigger GH secretion (26).

*b) Hyperfunction Suspicion: This is suspected* in a patient with a clinical presentation compatible with acromegaly or gigantism (if it is before epiphysis closure defined as a growth velocity for age > +2 SD or > p97 or a final height >+2 SD for the population) (27). This condition classically manifests as enlarged hands and feet, change in facial characteristics (square face with prominent features), separated teeth, mandibular malocclusion, carpal tunnel syndrome, asthenia, proximal myopathy, libido reduction, irregular menses, arterial hypertension with left ventricular hypertrophy. It may also be suspected when a pituitary adenoma is discovered (28).

### 4.2 Which Tests Should Be Requested?

- A serum insulin-like growth factor 1 (IGF-1) should be obtained to screen for suspected acromegaly. Of note, above-average serum IGF-1 concentrations are encountered during normal pregnancy, puberty and the post-pubertal period, whereas below average IGF1 levels are seen in uncontrolled diabetes or renal failure. Requesting growth hormone (GH) as the initial test is wrong because the levels of this hormone may fluctuate due to stress response (1, 29). If IGF-1 is positive, a GH suppression test is requested (GH measurement after 75g of glucose is administered).
- In case of acromegaly confirmation without a pituitary adenoma or pituitary hyperplasia, plasma growth hormone-releasing hormone (GHRH) must be requested to rule out a secreting neuroendocrine tumor. Additionally, a contrasted thorax and abdominal computed tomography and a somatostatin receptor scintigraphy must be performed to locate the source (29).

### 4.3 How Should These Tests Be Interpreted?

As previously mentioned, normal IGF-1 levels exclude an acromegaly diagnosis. In case of a result above the upper reference value, the diagnosis must be confirmed through a GH suppression test after an oral load of 75 gr of glucose. If no suppression occurs after the glucose load (levels > 1 ng/mL or > 0.4 ng/mL in ultrasensitive assays), the diagnosis is confirmed.

### 4.4 Main Mistakes in the Interpretation and Recommendations to Avoid Mistakes 

GH-producing adenomas, in rare cases, are big enough to produce large amount of hormone, so the "hook effect" may be observed when the number of plasma hormones overcomes the test detection capacity on the two-site immunoradiometric assay (IRMA) which could lead to a false interpretation of non-functional tumors. It is resolved by diluting the patient's plasma in 1:100, to process and then multiply the result for the conversion factor (2, 30).

The challenges of biochemical determination of IGF-1 are related to factors such as binding to transport proteins, use of different reference values, age variations, gender, estrogen effects (mainly peroral intake), and the number of persons involved to establish the reference values. To measure IGF-1 it's necessary to separate it from the binding proteins. Separation methods for these proteins include acidification followed by solid-phase chromatography with size exclusion, or ethanol-acid extraction.

Even though the results may properly correlate in healthy populations, the performance may vary under pathologic circumstances. In patients with diabetes mellitus, a frequent comorbidity in acromegaly, IGF-1 would be affected due to the higher proteolysis of IGF-1 binding protein 3 (IGFBP-3), one of the main binding proteins. At the same time, diabetes mellitus may lead to IGF-I glycosylation, and therefore being unrecognizable by monoclonal antibodies used in some assays. In cirrhotic patients, there is a decrease in liver GH receptors, therefore a decrease in serum levels of IGF-I and IGFBP-3 (31), which could lead to errors in the interpretation of these tests.

Another scenario is when seric IGF-1 increases are disproportionate to GH increase, which could be related to two reasons: GH secretion fluctuates more, and GH stimulates the secretion of both IGF-1 and IGFBP-3. In these cases where GH is disproportionate to IGF-1 levels, IGFBP-3 levels could be requested to further clarify this diagnostic challenge (32).

## 5 Gonadotropin Axis

### 5.1 When to Request a Gonadotropin Axis Test?

In men, the clinical manifestations of hypogonadism depend on the age of presentation. In the newborn, sexual ambiguity may appear if the deficit occurs in the organogenesis phase. Late in pregnancy, micropenis and undescended testicles could be observed. In the child and adolescent, the patient will present a delay in the pubertal development and testicles will have less than 6 ml of volume. In adults, signs and symptoms such as libido reduction, decrease in muscle mass and strength, erectile dysfunction, testicular volume reduction, infertility, and fractures in the absence of traumatism or osteoporosis will be observed (32).

These tests must be requested in any patient with a compatible clinical presentation. Additionally, screening is recommended for male patients who have received radiotherapy, pituitary surgical procedures, or those who have a diagnosis of pituitary adenoma, chronic opiates or chronic glucocorticoid treatment, and HIV patients with weight loss (33).

### 5.2 Which Tests Must Be Ordered?

- Total testosterone must be requested for male patients with the sample drawn between 8:00 and 10:00 hours due to circadian variation (which is lower in elderly patients) (34).
- To confirm the diagnosis, second total testosterone and sexual hormone-binding globulin (SHBG) should be requested to calculate free testosterone.
- Luteinizing hormone (LH) and follicle-stimulating hormone (FSH) must be requested to determine the origin.
- In the case of primary hypogonadism, karyotype must be requested to rule out Klinefelter syndrome.

### 5.3 How to Interpret These Tests?

In case the concentration is lower than the 2.5 percentile (p), i.e., 250-300 ng/dl, a second sample must be requested to confirm the diagnosis, associated with an SHBG test to calculate free testosterone (32, 35).

In case of a confirmed diagnosis with the second test lower than 2.5 p, FSH and LH should be requested. These tests are elevated in case of primary hypogonadism; if the tests results are normal or low, a hypothalamic or pituitary cause must be ruled out (36).

### 5.4 Main mistakes in the Interpretation and Recommendations to Avoid Mistakes

- Conditions that modify SHBG concentration must be taken into account (**Table 3**).
- Drawing sample in hours different than 8:00-10:00 hours
- Prednisone and other steroidal drugs interference. When the patient has systemic steroids intake, immunoassay interference may occur as it detects total testosterone levels, leading to falsely elevated measurements. When this phenomenon is suspected, liquid chromatography-mass spectrometry must be requested for an accurate measurement (3, 37).
- Requesting free testosterone measurement through a reference technique (equilibrium dialysis) is labor-intensive. "Bioavailable" testosterone measurement is commercially available – it is the addition of free concentration plus albumin-bound hormone (35, 36).

**Table 3:** Conditions that modify SHBG serum concentration.

Increase SHBG	Reduce SHBG
Thyrotoxicosis and hyperthyroidism	Hypothyroidism
Liver disease	Nephrotic syndrome
Estrogen and anticonvulsive drugs	Glucocorticoids, progestogen, androgenic steroids
HIV	Diabetes mellitus
Ageing	Acromegaly
Genetic polymorphism	Genetic polymorphism
Low weight or malnourishment	Obesity

Adapted from Ortiz-Flores et al. F. Assessment protocol of hypogonadism in adult men and the elderly. Medicine. 2020;13(32):1038 (34). HIV, Human immunodeficiency virus.

## 6 Thyrotropic Axis

### 6.1 When to Request a Thyrotropic Axis Test?

A thyrotropic axis test is requested when thyrotoxicosis or hypothyroidism are suspected. Hypothyroidism symptoms are usually subtle and include dry skin, cramps, cold intolerance, fatigue, and constipation. In more symptomatic cases, sleep apnea and carpal tunnel could occur. The most severe cases could present as myxedema coma (38). In thyrotoxicosis, symptoms include anxiety, sweating, hot skin, tremor, tachycardia (atrial fibrillation in the elderly), or weight loss (39).

Given the non-specific clinical presentation, the American Association of Clinical Endocrinologists (AACE) recommends thyroid-stimulating hormone (TSH) to be requested in the following scenarios:

- Type 1 diabetes mellitus, adrenal insufficiency, vitiligo
- Pernicious anemia
- First degree relative with autoimmune thyroid disease
- Neck radiation history, or radioactive iodine therapy
- Previous thyroid surgery
- Abnormal thyroid physical examination: nodule or goiter
- Mental disease
- Patients with amiodarone or lithium intake
- Dysmenorrhea, infertility, irregular menses
- Heart failure, hypertension and high cardiovascular risk patients (Type 2 Diabetes mellitus, e.g.).

### 6.2 Which Tests Must Be Ordered?

The test of choice in both thyrotoxicosis (whether it is due to hyperthyroidism or not) and hypothyroidism is TSH, which is measured through standardized immunoassays. Free T4 must be measured through standardized immunoassays as well (widely validated and less wasteful than equilibrium dialysis). TSH levels may be sensitive enough to rule out disease, but specificity increases when both tests are requested, and classifies the disease onto subclinical or overt.

T3 tests are less standardized and in most clinical scenarios do not offer changes in the final diagnosis nor the therapy, therefore, these tests are not routinely recommended. When thyrotoxicosis is present in the absence of elevated T4 or T4L, isolated T3 toxicosis is suspected and this test could be useful (40).

### 6.3 How to Interpret These Tests?

The American Association Of Clinical Endocrinologists (AACE) establishes the following diagnostic classification based on values TSH (41).

- In adults, if TSH greater than 10 mU/l with free T4 decreased, overt hypothyroidism is diagnosed. Severe hypothyroidism if TSH greater than 10 mU/l with decrease of free T4 and T3.
- Subclinical hypothyroidism: a) grade 1: TSH 4.5-9.9 mU/l with normal free T4 and b) grade 2: TSH equal to or greater than 10 with free T4 normal (42).
- In case TSH is above the upper reference value, but lower than 10 mUI/L, a diagnosis of subclinical hypothyroidism is performed; usually correlated with normal free T4 levels.
- In case of an elevated TSH value, associated with an elevated free T4 value, central hyperthyroidism or thyroid hormone resistance must be suspected.
- When TSH is normal or low, in a patient with low-free T4, central hypothyroidism must be suspected.
- If TSH is suppressed < 0,01 mUI/l and an increased free T4, overt thyrotoxicosis is the diagnosis, and thyroid scintigraphy must be requested.

### 6.4 Main Mistakes in the Interpretation and Recommendations to Avoid Mistakes

- TSH may vary up to 40% in the same individual during serial sampling as a circadian variation may be up to 50% on the same day. Therefore, the sample for TSH and free T4 must be taken from the same vein puncture. Some laboratories have protocols in case of an altered TSH result, leading to the automatic processing of a free T4 test.
- Levothyroxine therapy response must be monitored with TSH alone.
- TSH could be requested in a hospitalized patient only when thyroid disease is the cause of the hospitalization. It may be suppressed < 0,01 mUI/L in a critical patient or elevated up to 20 mUI/L after hospitalization in the recovery phase.
- Patients with pituitary adenoma and central hypothyroidism may have slightly elevated TSH values (6-7 mUI/L) due to secretion of biologically inactive hormone which is detected, nonetheless.
- Biotin interference: Multiple TSH immunoassays use non-competitive methods with biotin-streptavidin antibodies. These non-competitive "sandwich" assays suffer from interference when patients consume biotin, as streptavidin binds avidly to exogenous free biotin. After the wash step is performed and signal emission is evaluated through spectrophotometry, the sites that would bind TSH to form the sandwich will be occupied by biotin, limiting signal production, this lack of signal will be expressed as a falsely decreased TSH value (3).
- Free T4 and TSH receptor antibodies (TRAb): are determined with competitive immunoassay, in this technique results are inversely proportional to the number of free streptavidin residues when it’s performed, which will be filled with exogenous biotin, leading to a falsely elevated free T4 or TRAb. Therefore, for patients with a hyperthyroidism profile or even in suspected Graves’ disease (suppressed TSH, elevated free T4, and positive TRAb), if no correlation between clinical findings and laboratory results, biotin intake (voluntary or involuntary) must be ruled out.
- It is highlighted that the effect and extent of biotin interference are assay dependent, not only in thyroid function tests but also in multiple hormones (43). For this reason, result interpretation should be cautious in both clinical and laboratory setting.
- The presence of heterophile antibodies, usually human anti mice antibodies (HAMA), interfere with non-competitive immunoassay through binding of capture and signal antibodies, leading to a false signal which provokes an inappropriately high value (3, 39). This erroneous TSH elevation may lead to therapy adjustments in case of hypothyroidism; it must be suspected in cases of primary hypothyroidism with adequate adherence to levothyroxine and proper technique of intake, and when no clinical-laboratory correlation is observed.

## 7 Frequent Mistakes in the Evaluation of Miscellaneous Endocrine Tests

### 7.1 Renin-Angiotensin-Aldosterone System

The excess of aldosterone generates an increase in intravascular volume and promotes suppression of plasmatic renin activity which is associated with hypokalemia. A frequent mistake is to request aldosterone and renin levels in patients with potassium disorders. In hypokalemia, aldosterone release may be inhibited and falsely portray low values; on the contrary, if hyperkalemia was present, it induces falsely elevated aldosterone values (44).

Another frequent mistake is to evaluate these two hormones under the effect of multiple antihypertensive drugs. Antihypertensives, different to mineralocorticoid receptor antagonists (MRA) where possible, must be suspended at least two weeks before the tests are performed. Spironolactone and eplerenone must be suspended at least six weeks before. In patients with hard-to-control hypertension, it is possible to use alpha-blockers (like prazosin) or non-dihydropyridines calcium-channel blockers (like verapamil). If antihypertensives cannot be suspended, sample collection should be attempted with no diuretic use, and it should not be collected if MRA was not suspended six weeks prior. Estrogen or conjugated contraceptives intake must also be suspended for six weeks before collecting samples, due to elevated renin levels while using these medications (45).

### 7.2 Catecholamine Measurements and Its Metabolites

The adrenal medulla and the sympathetic paraganglia have chromaffin cells of neuroendocrine lineage, capable of synthesizing adrenalin (AD), noradrenaline (NA), and also dopamine. Catecholamines are either partially or totally converted into inactive metabolites (metanephrine and normetanephrine) by the catechol-o-methyltransferase. Catecholamine plasma release may be paroxysmal or minimal, which leads to the difficulty to detect elevated values in cases where pheochromocytoma/paraganglioma is suspected. The half-life of fractioned metanephrines is longer, as the intratumoral metabolism of catecholamines is performed independently from the excretion; therefore, its plasma liberation is mostly continuous (46).

A common mistake is to request catecholamine levels in ambulatory plasma, in patients with no overt paroxysmal crisis, which diminishes the sensitivity of this test (from 85%). Plasma free metanephrines have the best diagnostic performance (sensitivity 96-100%, specificity 89-98%), however, the readiness and time required to obtain a result in our field are complicated, hence, its recommended to request - in patients with proper renal function - 24-hour urinary fractioned metanephrines, as this study has better performance, with a sensitivity of 86-97% and a specificity of 86-95%. With the performance of these tests in mind, it is recommended to request metanephrines test over 24-hour urinary catecholamine tests and/or urinary vanillylmandelic acid, to avoid false negatives and to rationalize diagnostic tests requests (**Table 4**) (47).

**Table 4:** Situations and drugs and other substances that can interfere in the diagnostic study of pheochromocytoma.

**Drugs**
Anxiolytics, tricyclic antidepressants, and antipsychotics
Catecholamines and adrenergic agonists (including oxymetazoline: nasal decongestants)
Clonidine discontinuation
Amphetamines
Levodopa
Phenoxybenzamine
Beta-blockers
Buspirone
Hydralazine
Minoxidil
**Other substances:** Nicotine, caffeine, ethanol, cocaine
**Other situations:** Stress, advanced age and hypoglycemia

### 7.3 Bone and Mineral Metabolism

#### 7.3.1 Critically Ill Hypocalcemia

Patients undergoing critical care have a high prevalence of hypocalcemia. This is due to the inclusion of ionic calcium in arterial blood gas evaluation, or when ionograms include serum calcium. However, most of these cases of hypocalcemia will be asymptomatic and clinically irrelevant. Interventions must be considered only when hypocalcemia is coupled with symptoms, or ionic calcium less than 0.9 mmol/L (48).

#### 7.3.2 Parathormone Levels

A frequent mistake is to request PTH levels without specifying the assay generation, which must be a second-generation PTH test, or intact PTH (iPTH) levels. iPTH measurements must be validated for every laboratory, however, a repeated mistake is to assume that iPTH values are similar in patients with normal renal function and in those with an alteration of the glomerular filtration rate, where iPTH values can be elevated between three to ten times the reference values. This leads to a challenge in the interpretation of these scenarios. Therefore, the majority of endocrinologists and nephrologists' associations recommend taking into account an iPTH value greater than 300 pg/mL (49). Another frequent mistake when iPTH levels are being evaluated is in the context of hyperparathyroidism secondary to 25-OH vitamin D deficiency. As iPTH levels are elevated, calcium levels must be evaluated simultaneously. In case of normal or decreased levels, 25-OH vitamin D deficiency must be ruled out (50).

### 7.4 Glycated Hemoglobin 

Glycated hemoglobin is the product of the non-enzymatic glycation of hemoglobin A1. HbA1c is an accurate and specific test, which correlates with the mean glucose levels in the last 60-90 days. Nonetheless, this test does not take into account the glycemic variability, with the possibility of being normal in the context of high glycemic variability. Hence, a "normal" HbA1c may be obtained through multiple hyperglycemic or hypoglycemic values. In type 1 and type 2 diabetics with basal-bolus insulin therapy, a wrong interpretation of good metabolic control may be obtained if the HbA1c values are evaluated in isolation (51, 52).

When HbA1c is being interpreted, multiple conditions may lead to it being falsely low. These include hemoglobinopathies (Hb S, Hb D, methemoglobinemia), hemolysis, chronic lymphocytic leukemia, nitrates, drugs (dapsone, methylene blue, benzene derivates, vitamin C excess), hereditary spherocytosis, hemodialysis, phlebotomy, and posterior to blood transfusion. Among the conditions associated with falsely elevated HbA1c results, hemoglobinopathies of fetal Hb type, Hb D, carbaminohemoglobin, iron deficiency anemia, B9 or B12 vitamin deficiency. If one of these conditions is present in diabetic patients, it must be considered to avoid erroneous interpretation of HbA1c results (51).

### 7.5 Insulin Levels

When a patient is being evaluated for obesity, a common mistake is the evaluation of insulin resistance through a homeostatic model ("HOMA-IR") and the measurement of basal insulin. However, these two evaluations do not allow decision-making in a patient with obesity. The HOMA-IR value in different populations are not standardized and it is possible that a patient with overt acanthosis, elevated glucose, or HbA1c in prediabetes or diabetes range may show overt insulin resistance signs (53).

### 7.6 LDL Cholesterol Level Measurement in Hypertriglyceridemia 

In patients with elevated triglycerides, cLDL estimation may be wrong. In this scenario, it is useful to know that in cases where triglyceride levels are > 400mg/dL, serum levels of cLDL must be directly determined, instead of using the Friedewald formula [cLDL = total cholesterol – (cHDL + triglycerides/5)], which could lead to cLDL inaccuracies when triglycerides are elevated beyond those previously mentioned (54).

### 7.7 Chromogranin A Levels 

Chromogranin A (CgA) is a protein part of the granin family and is stored in the chromaffin granules. CgA blood levels are the universal marker of neuroendocrine tumors (NET). Higher levels are observed in metastatic carcinoid tumors (55, 56). CgA quantification is useful both in diagnosis, a follow-up to determine treatment response and recurrence or persistence of the disease. A study comparing three methods showed a sensitivity and specificity of 67% and 96% for immunoradiometric assay, 85% and 85% for enzyme-linked immunosorbent assay (ELISA), 93% and 85% for radioimmunoassay (RIA), suggesting that the best overall performance is for RIA. The most important challenge with CgA quantification is the lack of specificity, as is observed in **Table 5**.

**Table 5:** Factors that modify CgA concentration.

Factor	False-positive causes of CgA
Cardiovascular disease	Hypertension, heart failure, acute coronary syndrome.
Kidney disease	Altered kidney function / chronic kidney disease
Gastrointestinal tract disease	Chronic atrophic gastritis, inflammatory bowel disease, irritable bowel syndrome, pancreatitis, chronic hepatitis, cirrhosis
Non-neuroendocrine malignancies	Prostate cancer, ovarian cancer, breast cancer, colorectal cancer, pancreatic cancer, hepatocellular carcinoma, hematologic malignancies
Inflammatory disease	Rheumatoid arthritis, systemic lupus erythematosus, chronic obstructive pulmonary disease
Endocrine disease	Pheochromocytoma, hyperparathyroidism, hyperthyroidism, medullary thyroid cancer, pituitary tumors (excluding prolactinomas), hypercortisolemia
Drugs	Proton pump inhibitors (PPI), Histamine type 2 receptor antagonists (H2RA)
Other	Food intake or extenuating exercise before the test

Adapted from Gut P, Czarnywojtek A, Fischbach J, et al. Chromogranin A – unspecific neuroendocrine marker. Clinical utility and potential diagnostic pitfalls. Arch Med Sci. 2016 Feb 1; 12(1): 1–9 (55).

Proton pump inhibitors (PPI) therapy may increase CgA levels only five days after the first intake and leads to a CgA level five to10 times higher than the upper reference level. To avoid any impact of PPI therapy on CgA measurement, these drugs must be suspended at least seven days before the test. H2RA may influence CgA levels as well. It is suggested to suspend these drugs at least 24 hours before the scheduled CgA test (56).

Finally, **Table 6** summarizes the main disorders of the endocrine axes.

**Table 6:** Clinical presentation and hormonal evaluation of Hypothalamic–pituitary–adrenal axis. .

Pituitary axis	Clinical syndrome	Hormonal evaluation
**Lactotrope**	**Hyperfunction: hyperprolactinemia** Men: decreased libido, gynecomastia, erectile dysfunction, galactorrhea. Premenopausal women: oligomenorrhea or amenorrhea, decreased libido, galactorrhea. **Hypofunction: hypoprolactinemia** Men and non-pregnant women: no clinical syndrome. Pregnant women: inability to breastfeed.	**Hyperfunction** Baseline serum prolactin > 250 ng / dl: prolactinoma, exclude drugs (risperidone, sulpiride, haloperidol, metoclopramide, etc.) > 500 ng / dl: macroprolactinoma <100 ng / dl and macroadenoma (hook effect): sample dilution 1: 100 Polyethylene glycol (macroprolactin) precipitation: in hyperprolactinemia asymptomatic **Hypofunction** Baseline serum prolactin: low
**Somatotrope**	**Hyperfunction: acromegaly or gigantism (before closure of the epiphyses)** Increased size of the hands and feet, changes in facial features, hyperhidrosis, asthenia, carpal tunnel syndrome proximal myopathy, menstrual irregularity, decreased libido, hypertension, left ventricular hypertrophy, etc. **Hypofunction: GH deficiency** Adults (DGHA): increased fat mass, decreased lean body mass, osteopenia, dyslipidemia, insulin resistance and / or glucose intolerance	**Hyperfunction** Serum IGF-1 Normal levels: acromegaly / gigantism is excluded Elevated levels (for sex and age): OGTT (75 g) should be performed for GH. If there is lack of GH suppression to <1 ng / ml or <0.4 ng / ml with ultrasensitive assays, the diagnosis is confirmed **Hypofunction** Adults Serum IGF-1: In patients with more than 3 pituitary hormonal deficiencies, a low IGF-1 is diagnostic of DGHA. If there are <3 hormonal deficiencies and low IGF-1 levels, a stimulus test should be performed (≥ 2 abnormal tests confirm the diagnosis)
**Thyrotrope**	**Hyperfunction** Weight loss, palpitations, anxiety, heat intolerance, etc. **Hypofunction** Asthenia, weakness, weight gain, cold intolerance, constipation, dry skin, etc.	**Hyperfunction: secondary hyperthyroidism** Serum TSH: high or within the reference range LT4 and LT3: high A-GSU / TSH molar ratio: high Dynamic test for confirmatory diagnosis: no response to T3 suppression test and TRH test **Hypofunction: secondary hypothyroidism** Serum TSH: low or normal LT4: low
**Corticotrope**	**Hyperfunction: Cushing's syndrome** Specific data: proximal myopathy, skin atrophy, bruising spontaneous. Frequent symptoms: weight gain, depression, hirsutism, decreased libido, menstrual irregularity, etc. **Hypofunction** Common symptoms: fatigue, weakness, weight loss, nausea, vomiting, and diarrhea	**Hyperfunction** Screening tests *: UFC (at least 2 measurements), nocturnal salivary cortisol (2 measurements), TSD (1 mg), low-dose TSD for 2 days (2 mg / d for 48/h) Confirmatory tests: 2 mg-DST, nocturnal serum cortisol, CRH test, DEX-CRH test, DDAVP test, BPSC, other studies **Hypofunction** Baseline serum ACTH: low or normal Serum cortisol 8:00 am > 18 μg / dl: SAI is ruled out ** <3.6/5 μg / dl: SAI confirmed 3.6/5-18 μg / dl: Bump test recommended (TTI, 250 or 1 μg Synachten):> 18 μg / dl: ISS ** is ruled out, <3.6 / 5 μg / dl: confirmed SAI
**Gonadotrope**	**Hyperfunction** Premenopausal women: menstrual irregularity, infertility, galactorrhea, ovarian hyperstimulation syndrome Postmenopausal women: no clinical syndrome Men: testicular enlargement, hypogonadism **Hypofunction: hypogonadism** Men: gynecomastia, erectile dysfunction, decreased libido Women: amenorrhea / oligomenorrhea, decreased libido	**Hyperfunction** Serum FSH and LH: FSH within reference range or slightly elevated; LH suppressed or within reference range; Serum α-subunit and normal or elevated inhibin. Estradiol: elevated in premenopausal women Free and total serum testosterone: slightly below range baseline, normal or elevated **Hypofunction** Serum FSH and LH: low or normal Estradiol: low in premenopausal women (in postmenopausal women its measurement is not recommended routinely) Free and total testosterone: low

α-GSU, alpha subunit of glycoprotein hormones; BPSC, bilateral petrosal sinus catheterization; UFC, urinary free cortisol; DDAVP test, desmopressin test; DEX-CRH test, dexamethasone CRH test; DGHA, adult GH deficiency; LT4 and LT3, free T4 and T3; OGTT, oral glucose overload test; TSD, dexamethasone suppression test; TTI, insulin tolerance test. *Screening should only be performed if there is clinical evidence of suspected hypercortisolism **If an increase in CBG levels is not suspected. Adapted from Ramos-Levi et al. (57), Carlsen et al. (58), Beck-Peccoz et al. (59), Ntali et al. (60).

## 8 Conclusion

Endocrine laboratory tests are of vital importance, however, the knowledge of preanalytical, analytical, or post-analytical conditions, and an adequate interpretation, may determine the final interpretation of the result and avoid unwanted results for patients. This review is directed to medical students, general practitioners, and those unfamiliar with these common errors, to act correspondingly with the given information.

## Author Contributions

JA-P and LR-A contributed to conception and design of this review. JA-P wrote the first draft of the manuscript. JA-P, LR-A and JA-G wrote sections of the manuscript and revised the final draft. All authors contributed to the article and approved the submitted version.

## Conflict of Interest

The authors declare that the research was conducted in the absence of any commercial or financial relationships that could be construed as a potential conflict of interest.

## Publisher’s Note

All claims expressed in this article are solely those of the authors and do not necessarily represent those of their affiliated organizations, or those of the publisher, the editors and the reviewers. Any product that may be evaluated in this article, or claim that may be made by its manufacturer, is not guaranteed or endorsed by the publisher.
