# Corrigendum: Common pitfalls in the interpretation of endocrine tests

**DOI:** 10.3389/fendo.2022.875346

**Published:** 2022-11-09

**Authors:** Jose C. Alvarez-Payares, Jesus David Bello-Simanca, Edwin De Jesus De La Peña-Arrieta, Jose Emilio Agamez-Gomez, Jhon Edwar Garcia-Rueda, Amilkar Rodriguez-Arrieta, Luis Antonio Rodriguez-Arrieta

**Affiliations:** ^1^ Internal Medicine Department, Faculty of Medicine, University of Antioquia, Medellin, Colombia; ^2^ Internal Medicine Service, Institución Prestadora de Servicios (IPS) Universitaria - Clínica León XIII, Medellin, Colombia; ^3^ Faculty of Medicine, University of Antioquia, Medellin, Colombia; ^4^ Faculty of Medicine, University of Cartagena, Cartagena, Colombia; ^5^ Endocrinology Section, Internal Medicine Department, Faculty of Medicine, University of Antioquia, Medellin, Colombia

**Keywords:** endocrine test, hook effect, hyperprolactinemia, adrenal insufficiency, Cushing’s syndrome, acromegaly, hypogonadism

In the published article, there was an error in the legend for **Figure 1** as published. The image referenced from Haddad et al. image 3 of the Common Pitfalls in the Interpretation of Endocrine Tests, in this publication does not correspond to the hook effect. The **Figure 1** provided in this document, which is taken from the same reference as above, corresponds to Illustration of the high dose hook effect. The corrected legend appears below.

**Figure 1:**
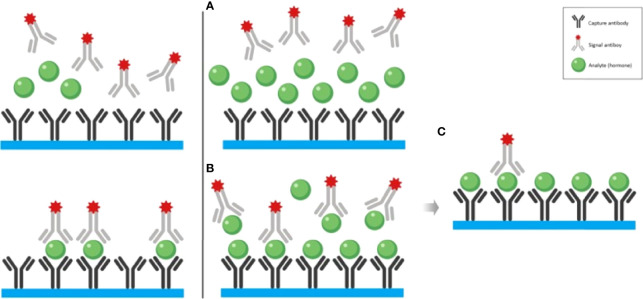
The left panel illustrates the non-competitive “sandwich” immunoassay with normal (or elevated within the tolerance of the assay kit) hormone concentration. The right panel illustrates the mechanism of the hook effect with exceedingly high hormone concentration. **(A)** At the sample that contains remarkably elevated hormone concentration is added to the test tube which contains both capture and signal antibodies. **(B)** It is both the capture and signal antibodies preventing the formation of the “sandwiches”. **(C)** After the washout phase, only a few “sandwiches” will be left producing a low signal. Adapted from Haddad et al. Clinical Diabetes and Endocrinology (2019) 5:12 (3) with previous authorization from the author (2).

The authors apologize for this error and state that this does not change the scientific conclusions of the article in any way. The original article has been updated.

## Publisher’s note

All claims expressed in this article are solely those of the authors and do not necessarily represent those of their affiliated organizations, or those of the publisher, the editors and the reviewers. Any product that may be evaluated in this article, or claim that may be made by its manufacturer, is not guaranteed or endorsed by the publisher.

